# Peripheral Embodiment: Polish Women Rebuilding Their Lives After Domestic Violence Through Their Bodies

**DOI:** 10.1177/10778012241275694

**Published:** 2024-09-02

**Authors:** Patrycja Sosnowska-Buxton, Ingunn Studsrød

**Affiliations:** 1 56627 The University of Stavanger, Stavanger, Norway

**Keywords:** Polish women, Catholicism, domestic violence, embodiment, recovery

## Abstract

In deeply Catholic Poland, domestic violence (DV) is often denied, downplayed, or justified, hindering its recognition as a pressing societal issue. This study addresses the scarcity of research on the experiences and recovery of Polish women from DV. Through feminist interviews with 13 women in Norway and Poland who survived DV, our findings reveal a complex entanglement of embodied experiences with history, religion, society, and gender hierarchies during their recovery processes. Participants emphasized the significance of “body works,” such as running and using makeup, as essential for empowerment and regaining control of their battered bodies and minds.

## Introduction and Background

Understanding the stories of Polish women who have experienced domestic violence (DV) is impossible without placing them within the context of Polish Catholicism (Grzyb, 2023). Thus, in this article, we explore how DV is an anathema (as religiously understood) to the sacred “natural” family (Graff & Korolczuk, 2021) and how enduring DV is expected of women by the Catholic Church and society in Poland (Kedzior, 2018; Korolczuk, 2009). Yet, contradictorily, being a victim, especially a female victim of DV is not revered but despised, akin to a weakling^1^ (in Polish *ofiara losu*; Sosnowska-Buxton, 2022). Framed as an individualized failure, women experiencing DV are accused by society as deserving the abuse or requiring it (Czarnecka, 2021) for as the Polish *humorous* rhyme goes “if a husband doesn’t beat his wife, her liver will rot.”

The female body occupies a special place in Catholicism (Clough, 2017; Sosnowska-Buxton, 2022). On the one hand, the female body is revered because of its ability to bear children. The Catholic view having children to be a woman's calling and her mystique as “the greatest God's work, which throughout human history have been accomplished in and through her” (Wojtyla, 1995, capitalization as in original). On the other hand, the female body is treated with suspicion because it simultaneously represents the feminine wiles embodied by Eve's tempting Adam into eating the apple. Her body is thus shameful and necessitates control, both publicly and in private by men, and their cultural, social, and political institutions (Clough, 2017).

Yet, paradoxically, Poland seems to be “a world without women” which means that women, their rights and needs are largely absent in public life, language (lack of, and resistance to, the usage of feminine nouns and verbs—Polish is a grammatical gender-specific language), law and politics (Graff, 2011). As such the female body is constructed as “merely” a vessel, a reproductive and familial utility kept inside, assuming her “natural” place in the home (Grzyb, 2018, 2023). Crucially, the Polish Catholic home is depicted as a safe haven. Church primates and Law and Justice politicians claim that DV does not occur in the home when the church's teachings are adhered (Gosc Niedzielny, 2018; Grevio Response Poland, 2021; Grzyb, 2020; Sosnowska-Buxton, 2022). However, DV in Poland is prevalent and some research currently places the rate of reported DV at 30% (Kantar Polska, 2019; Miedzik & Godlewska, 2014). The Kantar Report (2019) on DV in Poland notes that the occurrence and frequency of abuse is significantly underreported. Further, the report notes a lack of understanding of what constitutes DV as well as general societal permissiveness toward DV.

DV remains a political and ideological battleground in Poland. The Convention on Preventing and Combating Violence against Women and DV, also called the Istanbul Convention (IC), was signed in 2012 and ratified in 2015 by Poland. It is a major human rights legal instrument establishing comprehensive legal standards to ensure women's rights to be free from violence. It entails measures that need to be adopted by the signatory states. Yet, the Polish government threatened to withdraw from the IC on the grounds that it profoundly misunderstands the Polish Catholic family and is discriminatory against men because it privileges women (Grevio Response Poland, 2021).

Some significant legal changes have recently been implemented, such as changes to the legal definition from “violence in the family” to DV as well as mandated removal of abusers from home (Grzyb, 2018, 2020). However, DV legislation remains gender-neutral, conviction rates are very low, sentences are lenient and as Grzyb (2020, p. 164) notes “[t]here is a visible reluctance on the part of the criminal justice system to punish and correct domestic abusers.” Moreover, under an individualized model, DV abusers are portrayed as needing “help” to develop self-control and nonviolent conflict resolution (Dz. U. z 2021 r. poz. 1249 oraz z 2023 r. poz. 289).^2^ Importantly, there has been little state intervention or assistance historically offered to women and children experiencing DV and a chronic shortage of DV shelters in Poland (Grevio Poland, 2021).

Furthermore, Zielinska-Pocwiardowska and Sosnowska-Buxton (2023), argue that DV in Poland is profoundly misunderstood. They show that there is very little research on DV, especially sociological, and the available work tends to be superficial, laden with victim-blaming tropes (although notable exceptions include Urbańska, 2015 and Pokorna-Ignatowicz, 2014). Furthermore, prior studies describe DV as codependency between the abuser and the DV victim/survivor, and women experiencing DV are told to reassess their romantic partner choices so as not to attract abusers (Mazur, 2002; Młyński, 2012). Rajah and Osborn (2021, p. 1461) note that “there is limited comprehensive knowledge regarding how women's bodies and embodiment, that is, their physical and emotional practices and the cultural and social systems that influence them, figure in this process [resistance to intimate partner violence].” We also argue that there is little research on women's bodies and embodiment in recovering from DV and no research on the embodied experiences of Polish women.^3^

We undertook this research to better understand how Polish women living in Poland and Norway were rebuilding their lives following their experiences of DV. All Polish women in this research rejected the Polish version of the word “victim” and wished not to be written about as such, partly because of the word's profoundly negative connotations (see above) and partly because they felt that this word did not adequately describe their experiences. Moreover, there is no equivalent of “DV survivor”^4^ in Polish, the word is grammatically male and tends to be reserved for the survivors of the Holocaust^5^ (Sosnowska-Buxton, 2022). Thus, to honor these wishes as well as to challenge the dominant English-language way of naming DV experiences and to develop a way of speaking about the underrepresented voices^6^ (see Bradbury-Jones et al., 2019; Zielinska-Pocwiardowska & Sosnowska-Buxton, 2023), we use the descriptors the women used, such as “experienced DV,” “experienced intimate partner violence,” and/or “survived DV.”

In the interviews we overwhelmingly noted that women often referred to their female bodies,^7^ conceptualized both as an individual, embodied experience but also as a body living and engaging with a social world. In our analysis, we follow Tazzyman's (2020, p. 328) understanding of “embodiment as a concept [that] acknowledges both the physical materiality and the sociality of the body” or, what is felt through the material body is always mediated via social contexts in which the body is lived. This understanding, we argue, is particularly relevant in examining the Polish women's experiences of DV in the context of compulsory Catholicism (explained further on) in Poland. In this article, we analyze how women used and framed doing body works, for example, physical activities and makeup, as a means of rebuilding their lives after experiencing DV in a country steeped in Catholic church doctrine. First, we discuss the methodology that shaped our research project, explaining the sample, our methods, and our analytical lens. Secondly, we analyze how the women conceptualized the role, place, and expectations of the female body in Catholic Poland. Thirdly, we examine how doing “body works” embodies women's way of “recovering” from DV.

## Methodology

Feminist research methodology and practices focus on research as a process between participants and researchers, especially related to ethics (Hesse-Biber, 2012; Letherby, 2003). While we conducted semistructured interviews (the method) it is how we approached the before, during, and after the interviews as well as our framework for data analysis (the methodology) that are of key importance in a feminist research praxis (Hesse-Biber, 2012; Letherby, 2003). As feminist researchers, we recognize that the relationship between the researched and the researcher is complex and uneven. Moreover, we acknowledge that the subjectivity of the researchers shapes the process of knowledge production. Thus, in this section, we explain how we did the research.

In practical terms, we followed the standard Norwegian research ethics procedures (NESH, 2016) and our plan for handling data was approved by the Norwegian Agency for Shared Services in Education and Research.^8^ The information letters and consent forms were written in Polish. We emphasized informed consent, anonymity and that the women could withdraw from the project at any point. Additionally, our documents and our project emphasized our belief in the participants’ experiences of DV. In turn, this emphasis became a very important aspect of our research given that many women were suspicious of us and our motives. Sosnowska-Buxton (2016) has written about the barriers to accessing study participants, especially when researching sensitive and/or taboo topics, and trust is one of the key components, as was the case for this study. It took the lead author about 5 months to build relationships with potential participants, including talking on the phone and corresponding via emails before the women agreed to take part in the research. Moreover, the lead author engaged the help of *Intro*,^9^ a DV NGO in Poland, to help act as a critical gatekeeper to interviewees, by presenting the researcher as trustworthy and safe. Our working relationship continues to this day.

The lead author conducted all interviews with the Polish participants on Zoom, Teams, and WhatsApp. One interview was completed in person, and every participant did the interview from her home. All interviews were conducted in Polish and their lengths varied from 60 min to 2.5 hr. An additional written document was provided by one interviewee and three more participants continued sending WhatsApp messages with their thoughts about their recovery from DV. All women were offered anonymized transcribed interviews in Polish to review. Most women asked to see them, but none of the participants requested any changes.

The lead author transcribed and analyzed all data in Polish and approached the data analysis process in a constructivist grounded theory “style,” utilizing the constant comparative method (Glaser et al., 1968). As a feminist scholar with Polish roots, she also acknowledged her positionality, knowledges, and vested interests entering into the research project (Brown et al., 2011; Charmaz, 2008; Stanley & Wise, 1993). Once the data were coded, the lead author translated the relevant parts of the transcripts into English, emphasizing readability over literal translation and providing explanations of concepts which might be alien to non-Polish speakers.

Then, both of the authors started “thinking methodologically” in order to understand the interplay between “the methods used […] and the production and presentation of […] ‘findings’” (Letherby, 2003, p. 5). We applied a feminist sociological imagination to our knowledge production process, meaning that we considered the relationship between histories, biographies, and gender as well as religion to understand Polish society (Jackson, 2017; Letherby, 2018; Sosnowska-Buxton, 2025; Zielinska-Pocwiardowska & Sosnowska-Buxton, 2023). We agree with Jackson (1999, n.p.) that “a feminist sociological imagination enables us to see that personal troubles associated with gender(ed character of DV) are social in origin and hence potentially changeable.” Thus, for us as feminist scholars, it is important (our other vested interest) that our research shapes DV praxis that is sensitive to sociocultural specificities of Polish women's experiences which are often on the peripheries of scholars and policy-makers (Janion, 2009; Sosnowska-Buxton & Zielinska-Pocwiardowska, 2023; Zielinska-Pocwiardowska et al., 2020).

### Interview Sample

The lead author interviewed 13 Polish women between the ages of 18 and 59. The participants were White and heterosexual, and all women either were currently doing an undergraduate degree or already had a postgraduate degree, making this research sample unusual as only 26.9% of women in Poland have a university education as of 2021 (GUS, 2022b). All but two women were in full or part-time employment in white-collar jobs and two were registered disabled. Three women previously had experienced family violence at the hands of their fathers as children which continued up to their adulthood and two reported violence by their parents, including two women who experienced DV from their parents and former partners. Seven women experienced intimate partner violence, painting a complex multigenerational picture as DV is sometimes carried across familial relations. None of the interviewed women were in a relationship with their abusers. However, the women who had “joint” children still had to collaborate to some degree and coparent with the father of their children, and these women continued working with psychologists to protect themselves from coercive control. The women who experienced violence from their parents were no longer in any contact with them as a deliberate choice on the women's part.

All but one woman was raised Catholic and one was a Jehovah's Witness. At the time of the interview, four women declared that they were either an atheist or a nonbeliever, one was spiritual, three women were “a nonpracticing believer” (Polish *wierzaca nie praktykujaca—*an important denominator in Polish culture), three women described themselves as an ardent believer or deeply religious and of these, two were Catholic and one converted to Protestantism. Seven women lived in Poland and six lived in Norway.

## Findings

### The Female Body in Catholic Poland

Almost immediately at the start of the interviews, women talked about their religious views. All women, whether religious or not, talked about the female body being taboo, especially when it comes to its sexual functions because of the Christian beliefs they all grew up with. For example, Rosalia said:

just the issue of religion and that because of some things, because we were brought up with Christian or Catholic beliefs, and, you know, that masturbation is a sin, sex outside marriage is a sin, and sex, if you don't want to have children, is a sin. Another thing, contraception is, you know, (forbidden), a lot of taboo topics.

Rosalia talked about how she learned about the meaning of the female body through Christian beliefs and Catholic religious instructions in public schools that were repeated at home. The female body and its carnality are one of the many taboos in Catholic Poland. The female body is shameful, but it is also revered in Catholicism. On the one hand, the female body is pure, virginal, and maternal, therefore, sacred. On the other, it is profane because it leaks and is capable of sexual pleasure outside of procreation (Clough, 2017; Kieser, 2017). The woman is either “compliant and docile or transgressive and troublesome” (Inglis, 2003, p. 9). Later, Rosalia talked at length about how debilitating it was for her not knowing the vocabulary to describe her own body. She further described how important it was for her to learn this vocabulary as a means of recovery from DV and as a way to be assertive in gaining sexual pleasure. She even sent the lead author links to a Norwegian national TV program about puberty^10^ in which, to the participant's surprise the vocabulary and videos are explicit. The lack of knowledge of both proper and popular words for genitalia is noted as problematic in Poland where these words are seen as taboo.^11^

Drawing on Adrienne Rich's (1980) concept of compulsory heterosexuality, the lead author defines compulsory Catholicism as a process through which religion is also understood as a “natural” state of being, whereby everybody is Catholic and heterosexual and the two are intertwined accordingly with “natural” sexed roles. It renders other life philosophies, sexual orientations, and other differences invisible and actively silences them via social, legal, and political instruments. Whether the women grew up practicing Catholicism was irrelevant given the profound social, cultural, and political structures of compulsory Catholicism. As another participant, Aurelia said: “we are all Catholic, whether we are or not.” This is not surprising given that in 2018^12^ 91.9% of people in Poland identified as Catholic (GUS, 2022a). This status quo makes it very difficult to recognize that what one is experiencing is abuse and/or violence, especially if certain words and/or phrases for bodies and sexuality are taboo.

This lack of recognition of abuse was often mentioned in the interviews by Polish women living in Norway. For example, Rosalia shared: “I moved to Norway and I opened my eyes.” Aniela told the interviewer: “I didn’t know this was abuse until I came here (Norway) and started talking to other Norwegian mums.” Subordination and subjugation of women are deeply embedded in Polish society, and thus women often do not even identify with being victim-status and for them being dominated and marginalized is normalized (Graff, 2011). In comparison, none of the women living in Poland made a similar comment about recognizing their abuse. Rather, the women living in Poland described DV as codependency and a result of their own individual or psychological failures. For example, Jagna said that “it is obvious that I was codependent with my husband […] and I was sacrificing myself all the time.” Similarly, Mira said that when she married her abuser, she “was psychologically immature […] didn’t know my own boundaries nor those of my husband.” Both women agreed that it was reading psychological materials that helped them see that. This is not surprising given they had all previously talked about DV to priests and psychologists in Poland where this individualized narrative is pervasive (Zielinska-Pocwiardowska & Sosnowska-Buxton, 2023). However, Lena said that she used to be far harsher in her judgment of women who stayed with their abusers: “in the past I saw this (staying with the abuser) as an agreement to/permission to (violence) but not anymore. I now know it is very difficult to leave.”

Silence is deeply valued by Catholicism (Crisp, 2007) and thus there is very little to gain from talking openly about DV as it might be misconstrued as an attack on the Polish family, the church, and the country—what Sosnowska-Buxton (2022) refers to as the holy trinity. Here, we must clarify that by “talking openly” we mean talking to family and friends who may or may not tell others, or teachers and police, who have a statutory obligation to report DV. Talking to priests, especially considering the seal of confession, and therapists who are also bound by therapist–patient privilege are not talking openly but talking secretly. These two acts of speaking carry different weight and consequences. Talking openly might be considered an act of betrayal of the holy Polish trinity because DV becomes a public issue, whereas talking secretly helps solidify DV as a personal trouble rather than one of religion or patriarchy. It is a profound conundrum for Polish women to reconcile the mutually exclusive expectations of leaving a violent relationship because they need to break the “abuse chain” (Mazur, 2002) but at the same time fulfill their “natural” and patriotic duty to stay with their abuser. Therefore, many women endure the abuse in silence; neighbors do not ask about the abuse and women (and children) do not tell anyone and everybody goes to church every Sunday. This status quo is perfectly encapsulated by Lena who shared her experiences of witnessing DV at the hands of her father against her mother:

because on Sunday, of course, we all met in church, including my parents, and for me, everyone was just praying to god, and no one, no one (no neighbor who heard the abuse), responded to help my mother.

Lena's statement that everybody knew about the abuse but did nothing was mentioned in multiple interviews. Lack of help or intervention in family life is such a powerful phenomenon that it is referred to in Polish as *znieczulica*^13^ roughly meaning a structural anesthetized numbness to another's suffering (this is not simply the lack of empathy). Women frequently used this word during their interviews to describe the lack of reaction by neighbors or teachers. Social pressure to attend church, and pray to god while turning a blind eye to abuse despite knowing what is happening was commonly reported by the women.

Women further explained that the point of being a woman in Poland, a Catholic wife and a mother, is the expectation to endure *everything*. For example, Klara, who was sexually abused by her father, described how her mother's inaction to protect her and her family from abuse resided in this notion:

That it was violence, such (socio-cultural) pattern in Poland or not only in Poland, that if a woman gets married, she has to do everything. And she has to endure it, and even more. The Catholic approach is that if she's married, she made till the death do us part vow. At least that's how it is in my family and in other families, it's something we hear quite often. You have to stay here until the end.

Many women, like Klara, in the interviews also talked about the pervasive message that they heard in churches and at home that there is no escaping marriage or the fact that women have to “do everything.” Moreover, DV is an integral part of the sociocultural fabric of Poland. Like other women's narratives, Klara's clear understanding that to be a female body in Poland is to “endure it [DV], and even more” is remarkable, especially considering her very young age. Women's endurance of violence is sometimes regarded as a badge of honor in Poland because through it a woman can get close to Christ (Nowak & Ziemiński, 2023; Sosnowska-Buxton, 2022). In fact, Klara's mother decided to stay with her husband in the marital home and did not keep in touch with her.

All women who were ardent Catholic believers who either divorced (in civil court) or separated did not take communion but continued to go to church. Importantly, the women were reconciled with this as a price worth paying and were empowered by this choice. Interestingly, some women sought the blessing of a priest to get a divorce and in two cases the women received said blessing. This raises questions about how best to support Catholic women in leaving abusive relationships without undermining their belief in god, and how the Catholic church, including priests, can support women who have experienced DV and are seeking to leave their partners. It is evident from the interviews that some Catholic priests clearly disagree and disregard the church's teaching on this matter, and that other priests demand that they remain in abusive relationships.

The narratives from the interviews poignantly illustrate the profound impact of compulsory Catholicism on women's lived experiences, particularly in how they perceived and experienced their bodies in the context of DV. To initiate recovery from such violence, these women needed to fundamentally shift their engagement with personal agency. Engaging in physical activities emerged as a critical avenue for reclaiming their bodies and redefining their personal boundaries. In the following section, we explore how these practices or “body works” enabled women to regain control and assert their autonomy, marking a pivotal step in their journey toward healing.

### Body Works as a Recovery Tool From DV

The women experienced DV through the physical materiality of their female bodies which also lived a particular sociality (Tazzyman, 2020), that is compulsory Catholicism. This connection shows that personal troubles, like DV, are deeply interwoven with public issues such as religious and gender norms (Jackson, 2017; Letherby, 2018; Mills, 1959). The women were “embodied in social contexts,” deeply embedded within a network of social interactions with influential figures such as priests, therapists, and family members, as well as institutions like the church. These interactions facilitated a complex relational dynamic (Jackson & Scott, 2014, p. 578) that the women had to (re)negotiate in order to recover from DV. Through body works, that is physical activities such as running, yoga, or dance therapy and “feminine” behaviors and clothes, the women discovered a new vocabulary of self-expression and empowerment, transforming their bodies from sites of silent endurance to dynamic spaces of resistance and recovery. For example, Adela said

you have to fight with this whole cultural thing … just this kind of intimidation and it was affecting your psyche. […] So, this is especially the worst part—fear, because it is often this fear that paralyzes actions.

which shows how societal structures shape individual lives, constraining and enabling actions within these structures. All women had to “fight this whole cultural thing” which is a formidable constraint while recovering from DV. This fear that Adela mentions is sociologically significant as it not only reflects personal anxieties but also embodies the collective influence of societal expectations. It is particularly debilitating because it paralyzes action, maintaining the status quo by preventing dissent or deviation from accepted norms. In this section, we discuss how, through “body works,” the women fought this whole cultural thing, firstly by looking at physical activities and secondly at corporeal embodiment of femininity. We understand these practices not just as personal choices but shaped by and reactive to societal conditions, turning women's bodies from passive sites of suffering into active spaces of resistance and empowerment. This highlights a feminist sociological imagination's relevance in understanding the interplay between individual experiences and larger societal forces.

### Physical Activities as Connection and Empowerment

Herman (1997) notes that doing physical exercise aids recovery from trauma because it helps individuals regain a sense of control over/of their bodies and manage their stress levels. All women in the study were engaged in some form of physical activity, be it running or yoga. The effects were calming and empowering, as well as a way to reconnect with the corporal body. Interestingly, none of the women said that they exercised to look good. Rather, it was the stereotypically “feminine” behaviors, such as makeup and going to a beautician that were noted as tools to feel beautiful (discussed in the next section).

The women in our study engaged in physical activity both during DV, as well as after they left their abusive partners. For example, Aniela reported “I run. I started running so that I could get away from the abuse. I still run to keep my mind in check.” Initially, for Aniela, running was a coping mechanism, but also a physical act of taking herself out of the home where the abuse was happening. It was a temporary reprieve from the abuse. Aniela's sensate body (Jackson & Scott, 2014) was not subject to DV when she was running. Ida used running as a tool of recovery from DV and started it once she left her abuser: “I exercise (run) every day so that I can cope with what's happened to me.” It is telling that some women picked a solitary exercise like running which does not require social interactions with others, but instead focuses on the self. Being outside of the home and prioritizing their own needs, women engaged in exercise as an everyday transgressive act or “quiet subversions” (Beasley et al., 2012, p. 67). This is important for Polish women, who are culturally required to self-sacrifice and be confined to the home. Recognizing their own bodily sovereignty (Gotby, 2023) and exercising it, we would argue, is an important step in recovery from DV.

Physical activity continued to be an act of self-care and embodied empowerment during women's healing. On a physiological level, engaging in physical activities is widely acknowledged as a “feel good” process. Runner's high is a recognized phenomenon where the body releases endocannabinoids (Linden, n.d.) which are responsible for the feeling of calm. Furthermore, physical activity has been shown to have antidepressing properties and boost mood (Gammage et al., 2022). Perhaps it was this physiological effect of physical activity the women referred to as “keeping their minds in check.” While the physiological aspect of this process is critical, we also argue that it is a process that has changed women's relationship with their bodies and, thus, their social interactions. For example, Mira said:

Because it is through (physical) action that we express ourselves, and we express ourselves much more and perhaps most through action and less through speaking … It's a reconstruction. Appreciating a woman that she also does important things. Well, tough cookie, she can also be herself. It's a transformation. … I am strong. Yes.

As Sparkes and Smith (2012, p. 302) note, “the body is simultaneously cause, topic, and instrument of whatever story is told.” Importantly here, we would like to add that the body is also *how* the story is told.

For the women interviewed, engaging in physical activities was also a way of reconnecting to their bodies by feeling and experiencing their bodies. Finding or choosing a particular activity was not necessarily deliberate but incidental. For example, Pola said:

I’ve come across dance therapy, completely accidentally, and, and I remember this discovery that as though, you know I’ve started to feel my body, yup. […] It's hard to describe this experience, this difference, but I can only describe it this way: I started to feel my body as if it was here, as if it was a part of me, because before I had the impression that I was there, somewhere. Uh, I don't know. Well, my body simply serves me now. […] then I also started doing yoga.

Pola's quote suggests that before dance therapy she had not felt her body or had not felt it during the abuse years. As the women were repressing the “corporeal reality” (Kristeva, 1980/2023) of DV, exile from the self might have been a coping mechanism. Thus, the corporeal reality of physical activity might have been a way to reconnect with their bodies, to find and feel the self, or be in their bodies rather than it being a disembodied site of DV. For Pola, the body shifted from being the site of oppression, from being “there, somewhere” to the site of recovery, to being “here.” The material impact of physical activity and of reconnecting to the body was a significant aspect of “recovering” from DV. Pola's body became receptive to empowering sensations and experiences through physical movement.

A similar process of connecting back to the body from the “corporeal reality” of abuse was described by Ina, who said:

I was desperate not to feel, even for a minute, not to feel, my sensitivity was dimmed, I had nothing left to protect myself from the attacks. I had no energy. […] and then I went for a walk (she took up walking as an activity) and I started to feel, to feel … that I have a way out. Finally, I was living in agreement/accordance with myself.

Although it is tricky to discern from this quote Ina talked about how disconnected she was from her body and her “corporeal reality” when experiencing abuse. She was, in fact, “desperate not to feel” as a way to get a reprieve from abuse and all the negative sensations this brought. She also noted that this dimmed sensitivity was a double-edged sword as it also meant that she could not protect herself from the attacks. On the other hand, Ina also found her “way out” through her body. As Silverman (1996, p. 9) notes, “our experience of ‘self’ is always circumscribed by and derived from the body.” We thus argue that this was the case for Ina because her realizing that she had a way out was derived from her body walking. In this sense, she embodied her choices.

### Corporal Embodiment of Femininity as Empowerment

Feminist scholars have long critiqued patriarchal regimes to discipline female bodies through acts such as dieting, makeup, dress, and smiling in order to be perceived “feminine” and desirable (Alsop et al., 2002). However, in our study, women shared with us that they had to stop wearing makeup, stop smiling and not exercise because the female body was to be invisible and not desirable to other men. This was described as an expectation, rather than a betrayal of one's own body. Mira, for example, stated: “I’ve learnt that being a woman means not looking after yourself.” Thus, transgressing the expectation of not looking after oneself was a significant step in recovery from DV.

Kokkinou (2019) notes that makeup, for example, can be understood as a tool of oppression and empowerment of women, thus potentially a feminist act. For the women in our research, starting to wear makeup was empowering and a way to connect to “femininity” and their female bodies. Ida noted: “I started looking after myself. I cut my hair, put makeup on, had my nails done, I hadn’t done this for, for such a long time […]” because this made her feel “like a woman, womanly.” Later in the interview, she explained that being noticed by other men who saw her as an attractive woman was a huge boost to her self-esteem. However, realizing her ex-husband did not see her that way also made her upset. McCabe et al. (2020) argue that makeup application can boost women's confidence. Ida's quote also sheds light on the uncomfortable relationship between power and oppression of the female body in a patriarchal society where a woman's value is linked to her looks and carnal desirability. Empowerment may also be related to one's sexual desirability, and thus women may be simultaneously freed and trapped by her corporeal femininity (Andermahr et al., 2000). The female body, then, becomes a visible object in physical and social space as objectified female embodiment (Jackson & Scott, 2014). Tran et al. (2020) claim that wearing makeup can help women forge human connections. Our study participants seemed to have been deprived of that connection from other people, not just men, given deep cultural and religious ideals of embodying vessels of God's work.

The conditionality of the female body sovereignty—that is prioritizing emotional and material, here also bodily, needs of women (Gotby, 2023)—was encapsulated in the women's answer to what they mean by “femininity” or “womanly.” Lena explained it thus: “wearing makeup, dressing nicely and spending a lot of money on myself by buying clothes.” Makeup and nice clothes were linked by Lena to femininity and being/feeling womanly. This clearly shows that the women's choices regarding their bodily sovereignty were marked by gendered expectations and reinforced by them in symbolic and material ways (Gotby, 2023; Jackson & Scott, 2014; Morgan & Scott, 1993). Despite this conditionality, feeling like a woman by tending to oneself via cosmetics and clothes meant feeling empowered and being reconnected with one's body was important for the women recovering from DV. Whether this reconnection was stereotypically feminine or not, was in a sense irrelevant for women's recovery—at least at this particular point in time. Lena's decision to spend money on herself is another example of subversion by a woman prioritizing her needs. A similar sentiment was noted by Rosalia who said: “I am 100% important, my needs … everything I want and … Such nurturing, this return to femininity.” This is a powerful statement of recognizing and reclaiming the self and one's own needs. Rosalia makes an interesting connection between fulfilling her needs, nurturing, and returning to femininity. The female body and femininity are interconnected, and this “return” to femininity was conceptualized by the women as empowering and “a good thing” that they can finally feel like women rather than feeling oppressed.

DV had severed women's sense of femininity; they did not feel desirable as women and their battered and abused bodies appear to have made them feel worthless. For example, Julia said: “he told me I am stupid, stupid, and that our marriage would survive and he would also treat me better if I was prettier […] I was crushed.” The quote vividly illustrates embodiment being repeatedly called “stupid,” transcends speech to become an ingrained part of her self-image, demonstrating how abusive words can be embodied, deeply affecting one's self-worth and intelligence. Additionally, her husband's suggestion that their marriage's survival and his better treatment of her hinge on her being “prettier” highlights how gendered societal beauty standards can be weaponized against women. Julia's visceral reaction of feeling “crushed” is an embodied manifestation of profound emotional and physical impact of abuse. Another participant, Mira, noted:

I have already entered the model of a wife subordinated to her husband, that he is always right, that he knows better, that I should take care of him first, and never of myself … not at all. […] to be a woman is to be a shadow and … so my low self-esteem came out every now and then.

Adela also recalled that she felt “There's something wrong with me and I’m so alone …. I couldn’t shake it off, I’m not good enough.” The women's quotes show how a set of external expectations becomes a lived experience, influencing how women perceive their roles and value as an individual. The notion of being a “shadow” in Mira's narrative and Adela's feelings of being alone are a powerful embodiment of invisibility and insignificance, reflecting a deep internalization of being lesser than her male partner. Thus, the women, it would seem, confirmed Alsop et al. (2002) argument that not conforming to gendered beauty and behavior regimes *is* a betrayal of the female body.

## Conclusion

In this article, we examined the experiences of Polish women living in Poland and Norway who survived DV and were rebuilding their lives through their bodies. Our findings show that recovery from DV is a complex embodied process, entangled with religious and sociocultural determinants of compulsory Catholicims in Poland. The women's narratives revealed their clear understanding that the female body in Polish society is a vessel of God's work that has to endure abuse and violence and remain unheard and unseen. Thus, an important transgression of norms was necessary for women to begin the recovery process, where the body changed from being the site of oppression to a site of empowerment. The women deployed two embodied tools—what we called “body works”—namely doing physical exercises, such as running or dancing, and engaging in “feminine” behaviors such as applying makeup and wearing nice clothes. These “quiet subversions” of Catholic gendered social orders required bravery and determination on the women's part but also, perhaps inadvertently, led to reinforcing other patriarchal norms ordering the female body. Importantly, this negation and then reinforcement of gendered norms was seen by the women as empowering and enabling them to feel whole. The women's experiences clearly demonstrate that the body is not just inhabited but also lived, shaped by and experienced through social and corporeal interactions between the self and society. As Polish women's voices remain largely absent in scholarship on the interconnections of religion, culture, and DV, this article is a much-needed intervention, and in our view, the start of a much longer engagement in the interconnections of religion, culture, violence, and embodied empowerment.

## Notes

1. *Ofiara losu* as well as *ofiara* have profoundly negative social connotations in the Polish language and society. It is one of the reasons why in DV practice the word is rarely used, although it is used in law and policy.

2. The profound misunderstanding of DV as a lack of self-control in Polish DV prevention law is troubling but reflects social and political “approaches” to combating DV. The law also hints at a societal perception that DV *is* another way of conflict resolution in Polish society.

3. Zielinska-Pocwiardowska and Sosnowska-Buxton (2023) note the body as a potential new area of Polish sociological research on DV, especially in the context of taboo.

4. There are some attempts to create neologisms such as “przetrwanka” or “ocalenka” but these are not used in common parlance.

5. https://sjp.pwn.pl/poradnia/haslo/Ocalaly-ocalony-ocaleniec;17780.html.

6. For further discussion on the lack of Polish vocabulary to talk about DV see Sosnowska-Buxton (2022), Zielinska-Pocwiardowska and Sosnowska-Buxton (2023), Sosnowska-Buxton and Zielinska-Pocwiardowska (2023).

7. When we use “female body” we apply the meaning that the interviewed women conveyed but biological essentialism, neoliberal and patriarchal assumptions seemed to underpin the women's conceptualization of the “female body.”

8. Processing of personal data was approved on November 29, 2021, reference number: 771023.

9. https://intro.org.pl/odwiklane/.

10. https://nrksuper.no/serie/newton-pubertet/DMPV74100815/sesong-1/episode-8.

11. According to a recent survey, 26% of Polish women cannot name their reproductive organs (Spacecamp for AA Oceanic, June 2023).

12. Data from the 2021 Census are still being analyzed.

13. https://sjp.pwn.pl/slowniki/znieczulica.html.
